# Termiticidal, biochemical, and morpho-histological effects of botanical based nanoemulsion against a subterranean termite, *Odontotermes Formosanus* Shiraki

**DOI:** 10.3389/fpls.2023.1292272

**Published:** 2024-01-08

**Authors:** Raghda Nasser, Ezzeldin Ibrahim, Hatem Fouad, Farhan Ahmad, Wuhan Li, Qihuan Zhou, Ting Yu, Nooney Chidwala, Jianchu Mo

**Affiliations:** ^1^ Ministry of Agriculture Key Lab of Molecular Biology of Crop Pathogens and Insect Pests, Key Laboratory of Biology of Crop Pathogens and Insects of Zhejiang Province, Institute of Insect Sciences, College of Agricultural and Biotechnology, Zhejiang University, Hangzhou, China; ^2^ Zoology and Entomology Department, Faculty of Science, Minia University, El-Minia, Egypt; ^3^ State Key Laboratory of Rice Biology and Breeding, Ministry of Agriculture Key Laboratory of Molecular Biology of Crop Pathogens and Insects, Key Laboratory of Biology of Crop Pathogens and Insects of Zhejiang Province, Institute of Biotechnology, Zhejiang University, Hangzhou, China; ^4^ Department of Vegetable Diseases Research, Plant Pathology Research Institute, Agriculture Research Centre, Giza, Egypt; ^5^ Department of Field Crop Pests, Plant Protection Research Institute, Agricultural Research Centre, Cairo, Egypt; ^6^ Entomology Section, Central Cotton Research Institute, Multan, Pakistan

**Keywords:** nanoemulsion, eucalyptus oil, nutmeg oil, *Odontotermes formosanus*, termiticidal, biochemical, morpho-histological

## Abstract

Recently, the use of nanopesticides has shown significant efficacy in the control of many pests. However, the effect of nanopesticides, especially nanoemulsions, on suppressing termites, *Odontotermes formosanus* (Shiraki, 1909) (*O. formosanus*), has not been studied yet. Therefore, this study aimed to produce nanoemulsions of the essential oils of eucalyptus (*Eucalyptus globulus* Labill; E-EO) and nutmeg (*Myristica fragrans* Houtt; N-EO) to suppress *O. formosanus*. The analysis of eucalyptus nanoemulsion (E-NE) and nutmeg nanoemulsion (N-NE) was confirmed by using UV-Vis, dynamic light scattering, zeta potential, transmission electron microscopy, scanning electron microscopy, and energy dispersive spectroscopy. In addition, chemical analysis by Gas Chromatography with a mass spectrometer (GC-MS) exhibited the major constituents of E-NE and N-NE. The principal chemical components of E-NE included D-limonene, eucalyptol, 1,5-cyclooctadiene,3,4-dimethyl, benzene, and 1-methyl-3-(1 methylethyl)-, while the main constituents in N-NE were cyclohexane,1-methylene-4-(1 methylethenyl)-, eucalyptol, and L-. alpha. -terpineol. The mortality rates were 100% and 99.53%, respectively, after 24 hours of treatment with a concentration of 140 mg/mL, compared to 23.43% and 43.55%, respectively, from E-EO and N-EO treatment. These results refer to the essential oils’ nanoemulsion as far more effective than the essential oils themselves. Furthermore, the effects of E-NE and N-NE on detoxification enzymes such as acetylcholinesterase, carboxylesterase, acid and alkaline phosphatase were investigated, as well as total protein concentrations, and the results have been found to be significantly increasing or decreasing in comparison with control. Besides, histological and morphological alterations found post exposure to E-NE and N-NE were shown. Overall, the results from this study clearly indicate that the nanopesticide-formulated nanoemulsions may have great potential to be used as novel, environmentally safe insecticides for controlling *O. formosanus*.

## 1 Introduction

Termites are ancient social insects that feed on cellulose, causing great damage, especially to trees, buildings, and dams. Depending on their feeding habitat and ecology, they are divided into three divisions: subterranean termites, dry wood termites, and damp wood termites. Subterranean termites are among the most dangerous, with damage control and repair costing more than 230$ million. In addition, more than 70% of the damage to facilities is caused by it (Rust and Su, 2012). In China, the black-winged termite, *Odontotermes formosanus* (Shiraki, 1909) (*O. formosanus*), is a serious pest because it threatens water storage facilities by moving large amounts of soil needed for nesting, thereby creating subsurface cavities within dams (Xu et al., 2010). This termite creates tunnels of mud and coating that encase the tree trunks, where it devours bark and phloem, eventually killing the tree. This is a serious problem with seedlings in plantations, forests, and urban green spaces (Dodji Kasseney et al., 2011). In addition, some of its members may spread plant pathogens from diseased trees to healthy ones, indirectly increasing the damage done (Puche and Su, 2001). Currently, chemical pesticides are the main method in the fight against termites. However, the long-term excessive use and widespread application resulted in their danger to the environment and human life (Mardiningsih and Rizal, 2022). Therefore, it is necessary to search for a new strategy that is more effective in suppressing this pest, stopping its harm, and being safe for the biological system, other organisms, and humans.

Among these strategies, botanical derivatives could be the right choice because many of the active ingredients have protective abilities against a lot of biotic agents, including major pest species. Essential oils (EOs) have attracted the attention of many researchers as a result of their effectiveness against many harmful pests (Giunti et al., 2019; Giunti et al., 2022). For example, eucalyptus essential oil (*Eucalyptus globulus* Labill) (*E. globulus*) and nutmeg essential oil (*Myristica fragrans* Houtt) (*M. fragrans*) are known for their biological and pharmaceutical properties. These oils contain chemicals with antioxidant, antibacterial, and insecticidal effects (Mssillou et al., 2022). Eucalyptus oil (EO) has been demonstrated to be an extremely effective insecticide against a variety of storage pests, including *Tribolium castaneum* (Herbst, 1797) (*T. castaneum*), *Sitophilus oryzae* (Linnaeus, 1763) (*S. oryzae*), *Rhyzopertha dominica* (Fabricius, 1792), *Sitophilus zeamais* (Motschulsky, 1855), and others (Lee et al., 2004; Mossi et al., 2011). Furthermore, eucalyptus oil has the potential to function as a natural insect repellent to ward off pests like mosquitoes, poisonous arthropods, and other herbivores (Batish et al., 2008). Additionally, *T. castaneum*, whitefly adults and nymphs, *Lymantria dispar* (Linnaeus, 1758) larvae, *Callosobruchus maculatus* (Fabricius, 1775), *Chrysomya albiceps* (Wiedemann, 1819), and *Musca domestica* (Linnaeus, 1758) larvae were all inhibited by nutmeg essential oil (Adedire, 2002; Kostić et al., 2013; Wagan et al., 2017; Gao et al., 2020; Cossetin et al., 2021). However, its application faces some limitations, such as its poor water solubility, rapid decomposition, high volatility, and combustibility, which hinder its direct use in field conditions. Therefore, there is a need to provide it stably and more effectively and help solve the problems mentioned above (Laudani et al., 2022).

Nanoemulsion is one of the most important ways to overcome the limitations faced by essential oils when applied and taken advantage of their significant role as a safe alternative to pesticides (Chen et al., 2020). Nanoemulsion preparations require mixing two immiscible liquids, such as water and oil, in addition to an emulsifying agent to reduce the surface tension between oil and water, which results in spherical nanodroplets with a transparent or opaque appearance and sizes ranging from 1 to 100 nanometers (Sheth et al., 2020; Kumar et al., 2021). Apart from surfactans and cosurfactans, there are two ways that rely on energy to produce quality nanoemulsions. Both high- and low-emulsification energy techniques can be used to create nanoemulsions (Jasmina et al., 2017). High-energy methods make use of mechanical apparatus (such as high-pressure homogenizers, ultrasonic techniques, or microfluidizers) that generate strong forces that can form minuscule oil droplets. Low-energy techniques rely on the compositional features of the system and include intricate interfacial hydrodynamic phenomena. Solvent displacement, phase inversion, and spontaneous emulsification are examples of low-energy processes (Yadav et al., 2020; Santamaría et al., 2023). Therefore, this study aimed to prepare nanoemulsions from eucalyptus essential oil of *E. globulus* and nutmeg essential oil of *M. fragrans* using a high-speed homogenizer and study their characteristics and mechanism of action to suppress *O. formosanus*.

## 2 Materials and methods

### 2.1 Materials

Eucalyptus essential oil of *E. globulus* and nutmeg essential oil of *M. fragrans* were purchased from Jiangxi Medicinal Oil Refinery Factory, South China, and Tween 80 (Polyoxyethylene 20 monooleate) was purchased from Sigma Aldrich Chemical Corporation, China. Bradford protein, acetylcholinesterase, carboxylesterase, alkaline phosphatase, and acid phosphatase assay kits were purchased from Nanjing Jiancheng Bioengineering Institute, China; all other chemicals, and reagents used were of the highest analytical grade and purchased from local companies. Ultrapure water was obtained from YJD-UPWS Ultra-Pure Water System, Technology Co., Ltd, China, with a resistivity not less than, 18.2MΩ.cm, was used for the preparation of all solutions.

### 2.2 Termite colonies feeding and sample collection

Four colonies of *O. formosanus* (height 6-8 cm; diameter 6-12 cm) harboring king (s) and queen (s) were collected from a forested area of Hangzhou City, Zhejiang Province, China (N 30°18´, E 120°50´). The colonies along with fungus-combs were wrapped in plastic film separately and transported to the lab within 12 h of excavation. All colonies were placed separately in plastic chambers (45 cm length × 45 cm width ×30 cm depth) containing clay soil obtained from the area where the colonies were collected. The rearing systems were maintained in complete darkness at 26 ± 1°C and > 90% relative humidity. After the colonies became stable in a laboratory, they were allowed to feed on sweet osmanthus (*Osmanthus fragrans* Lour) (*O. fragrans*).

### 2.3 Preparation of nanoemulsions

The preparation of eucalyptus nanoemulsion (E-NE) and nutmeg nanoemulsion (N-NE) from eucalyptus essential oil (E-EO) and nutmeg essential oil (N-EO), respectively, was performed according to the method of Rodríguez-Burneo et al. (2017), with short modifications. In a nutshell, the oil-water nanoemulsions were prepared using essential oils (14%, v/v), ethanol, (3%, v/v) and Tween 80 (3%, v/v). These components of the oily phase were vigorously mixed for 5 min at 15000 rpm with a magnetic stirrer (RCT digital hotplate stirrer). The 20% of prepared oily phase was combined with distilled water (aqueous phase), while continuously stirring magnetically for 15 minutes to obtain the final volume of 100%. To develop the coarse emulsions, further homogenize to be done for 3 min at 16,000 rpm using a high-speed homogenizer. To avoid evaporation and successfully create nanoemulsions, the previously prepared coarse emulsions were placed in an ice bath during ultrasonication process. The coarse emulsions were sonicated using a probe sonicator (Sonics & Materials Inc., U.S.A.) with a diameter of 6 mm at 75% of full power amplitude (75 W) for 20 min. For further work, the nanoemulsion was kept in opaque bottles at room temperature.

### 2.4 Characterization of nanoemulsions

Several techniques were used to characterize E-NE and N-NE according to the method of Ibrahium et al. (2022), with a few modifications. The surface plasma resonance of E-NE and N-NE was measured by ultraviolet using UV-Vis spectrophotometry (Shimadzu Corporation, Kyoto, Japan) in the range of 200-800 nm. The average droplet size and size distribution (polydispersity index, PDI) were measured using dynamic light scattering (DLS), and the stability of E-NE and N-NE under an electric field was estimated through zeta potential (Z-sizer Nano, Malvern Instruments) at 25°C. To avoid multiple scattering effects, 0.5 mL of the prepared NE was diluted in 99.5 mL of double-distilled water. Additionally, the shape and size of the drops of the E-NE and N-NE were recorded by placing one drop of the sample on a carbon-coated copper grid which was negatively stained with 2% phosphotungstic acid for 24 h to form a thin film, and then the sample’s structure was observed by using TEM (JEM 1230, JEOL, Tokyo, Japan) and SEM (SEM, TM 1000, Hitachi, Japan). The energy dispersive spectrum (EDS) was employed to confirm the presence of metals in E-NE and N-NE. The stability of the nanoemulsion was evaluated at room temperature by centrifuging the produced NEs at 10,000 rpm for 20 min; additionally, 20 mL of NEs were kept at four different temperatures: -4°C, 20°C, and 45°C, and their phase separation and creaming were monitored.

### 2.5 Chemical characterization of nanoemulsions

GC/MS analyses of E-NE and N-NE were performed with Gas Chromatographs instrument (7890B, Agilent, USA), coupled with a mass spectrometer detector (7000C, Agilent, USA). The GC-MS system was a DB-5MS column (30 m by 0.32 mm i.d., 0.25 um film thickness). The analytical conditions were carried out using helium as carrier gas at a flow rate of 1 mL/min, injection, 0.2 µL (1:10% hexane solution); and a split ratio of 1:10 using the following temperature program: 60°C for 4 min, rising at 10°C/min to 300°C and holding for 5 min. The injector and detector were held at 250°C. Diluted samples (1:10 hexane, v/v) of 1 microliter of the mixtures were always injected. The identification of chemicals was based on the comparison between the pure chemicals of the retention times (RTs) of the compounds and their linear retention indices (LRIs), as well as computer matching against commercial (NIST 05, Wiley FFNSC, and ADAMS) and homemade libraries (Masada, 1976; Davies, 1990; Adams, 2017). The relative percentage of compounds was estimated by normalizing peak areas (Van Den Dool and Kratz, 1963).

### 2.6 Termiticidal assay

The toxicity of six concentrations (4.37, 8.75, 17.5, 35, 70, and 140 mg/mL) of nanoemulsions (NEs) and essential oils (EOs) of eucalyptus and nutmeg against *O. formosanus* old workers was determined according to the methods of Femi-Ola et al. (2008), with a few modifications. Sterile circular-shaped filter papers were saturated with 0.5 mL of the previously indicated concentrations of NEs and EOs. The treated papers were left to dry at ambient temperature and then put into the Petri dishes (6 cm in diameter) at the rate of one paper per dish. Filter papers saturated with 0.5 mL of Tween 80, ethanol, and double distilled water (ddH_2_O) were enrolled as a control. Ten old workers of *O. formosanus* for each dish were put in. Which were allowed to feed on preground *O. fragrans* mixed with the same treatment. The plates were incubated under a constant condition (27 ± 1°C, RH 60 ± 5%, L: D = 16 h: 8 h). The experiment was repeated twice with three replications. The termiticidal effect of NEs and EOs was monitored by recording mortality from 3 h to 96 h after treatments, and the LC_50_ and LC_90_ were calculated. The dead workers were identified when they failed to move after being probed with a small brush. With no appendage movement, the test workers were considered dead.

### 2.7 Biochemical assay

#### 2.7.1 Preparation of whole body homogenates

According to the method of Fouad et al. (2021), with a few modifications, the whole body homogenates of *O. formosanus* from old workers was prepared. In brief, the workers treated and untreated with LC_50_ and LC_90_ of E-NE and N-NE for 24 h were homogenized in Eppendorf tubes (held in crushed ice) using a Teflon hand homogenizer in 1 mL of 0.9% saline. The homogenates were centrifuged at 12000 rpm for 20 min at 4°C, and the clear supernatants were collected and stored at -80°C for use in biochemical assays, which was detected using commercial assay kits following to the manufacturer’s instructions. All biochemical tests were done for three repetitions. The solution for homogenization and glassware were kept at 4°C before use, and the homogenates were hung on ice before further investigation.

#### 2.7.2 Determination of protein concentration

The Bradford assay was used to determine the total protein content of the preparations by the Coomassie Brilliant Blue G-250 dye-binding method (Bradford, 1976), using bovine serum albumin (BSA) as the standard. The OD value was recorded at 595 nm using a 96-well microplate reader.

#### 2.7.3 Acetylcholinesterase activity

Acetylcholinesterase (AChE) activity was measured using the colorimetric method (Ikezawa and Taguchi, 1981), based on the fact that AChE catalyzes the hydrolysis of acetylcholine to produce choline, and choline reacts with disulfide p-nitrobenzoic acid (DTNB) to produce 5-mercapto-nitrobenzoic acid (TNB). The OD value was measured at 412 nm using a 96-well microplate reader. The enzyme activity was defined as 1 nmol of TNB substrate decomposing in the hydrolysis reaction per mg of protein, and the activity was reported as nmol/min/mg prot.

#### 2.7.4 Carboxylesterase activity

Carboxylesterase (CarE) activity was detected using the colorimetric method (Teese et al., 2013), based on the fact that CarE catalyzes the formation of naphthol by acetate-1-naphthyl ester, which further reacts with the solid blue color developer to form a colored substance. The OD value was detected at 450 nm using a 96-well microplate reader, and the enzyme activity was reported as nmol/min/mg prot.

#### 2.7.5 Alkaline and acid phosphatase activity

ALKP and ACP activities were performed using the colorimetric method (Li et al., 2017), based on the hydrolyze para-nitrophenyl phosphate chromogenic substrate, to produce 1 micromole of p-nitrophenyl per minute. The absorbance was read at 405 nm, and the results were expressed as U/ml protein.

### 2.8 Morphological observations post-exposure to nanoemulsions

According to the method described by Neves Filho et al. (2009), with slight modifications, the effects of LC_50_ and LC_90_ of E-NE and N-NE on the morphological changes of *O. formosanus* old workers were investigated. Briefly, the treated and untreated workers, with LC_50_ and LC_90_ of E-NE and N-NE for 24 h, were gently collected and washed with ddH_2_O twice. Samples were immediately soaked overnight in 2.5% glutaraldehyde and then washed twice with phosphate buffer saline (PBS) before being put in osmium tetroxide for one hour. The samples were washed again with PBS, dehydrated in a graded ethanol series, and then the samples were dried by using the critical point dryer, subsequently were spurted with 45 nm gold powders, attached to the stubs, and examined under a scanning electron microscope (TM-1000, Hitachi, Japan).

### 2.9 Histological observations post-exposure to nanoemulsions

The histological observations in old workers of *O. formosanus* post-exposure to E-NE and N-NE for 24 h at LC_50_ and LC_90_ were carried out following the procedure by Al-Mehmadi and Al-Khalaf (2010) with minor changes. Briefly, the adult workers were fixed in 4% paraformaldehyde solution for 48 h, dehydrated using a graded series of ethanol, and cleared with xylene solution. Next, they were embedded in paraffin blocks using melted paraffin at the embedding station. The paraffin blocks were cut into 5-μm-thick sections using a rotary microtome and stained with hematoxylin and eosin. The glass slides were examined for abnormalities under a light microscope (Leica DM500 China).

### 2.10 Statistical analysis

The percentage of mortality data was subjected to probit analysis, and LC_50_ and LC_90_ were calculated statistics with 95% confidence limits for lower and upper values. For evaluating the biological variables observed in the experiments, data were analyzed using one-way analysis of variance (ANOVA), followed by Tukey’s HSD test using the software SPSS version 21 (SPSS Inc., Chicago, IL, USA). In all data analyses, a P-value < 0.05 was considered statistically significant to assess the differences among the control and treated groups.

## 3 Results

### 3.1 Characterization of nanoemulsions

**Figure 1:**
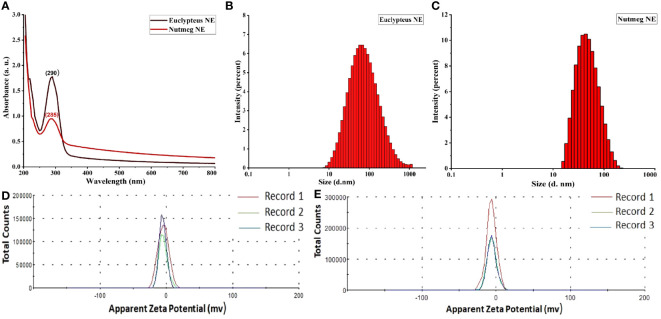
Characterization of prepared nanoemulsions. **(A)** UV-Vis spectrophotometers peaks of eucalyptus and nutmeg NEs; **(B)** Size distribution of E-NE; **(C)** Size distribution of N-NE; **(D)** Zeta potential distribution of E-NE; **(E)** Zeta potential distribution of N-NE. Records 1-3 represent repetitions of the experiment.

**Figure 2:**
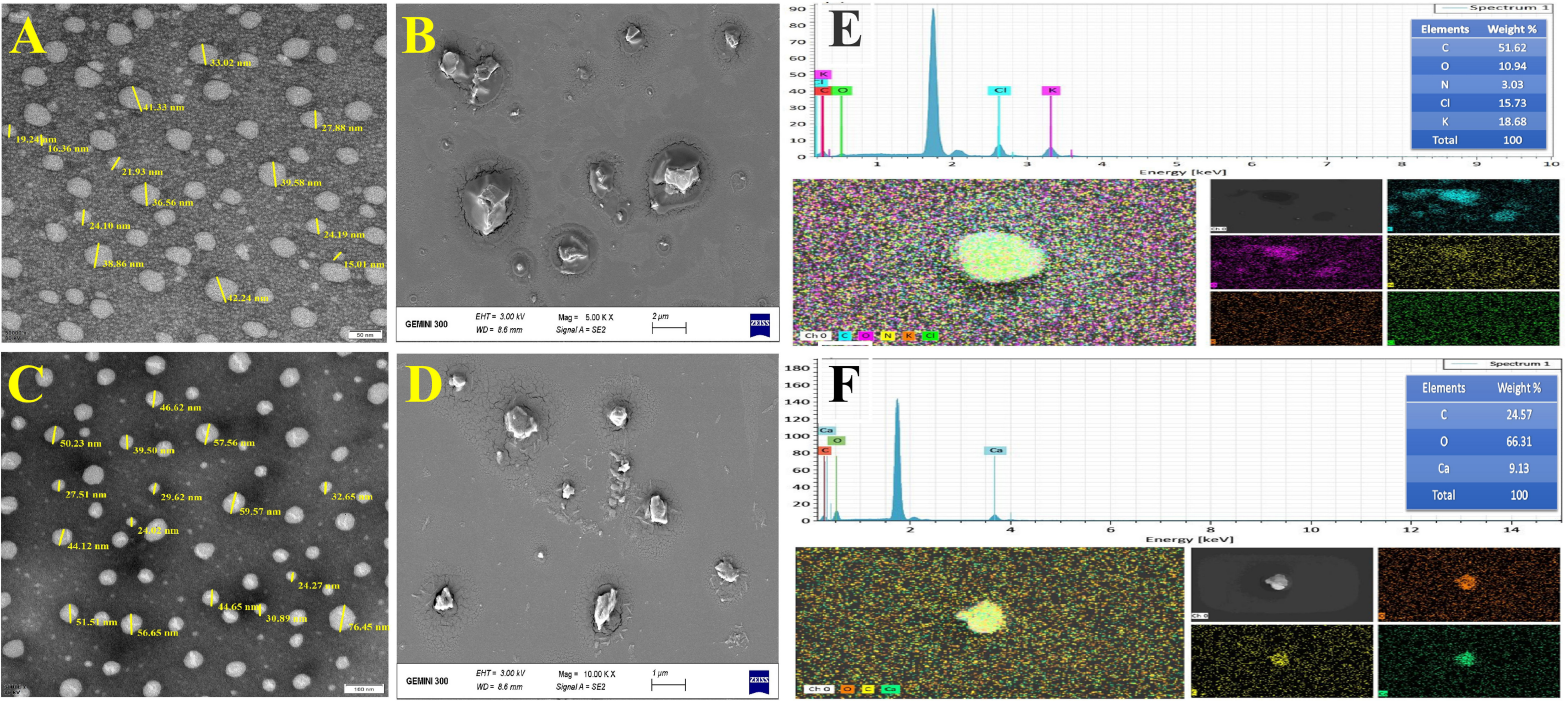
Characterization of prepared nanoemulsions. **(A)** Transmission electron microscopy of E-NE; **(B)** Scanning electron microscopy of E-NE; **(C)** Transmission electron microscopy of N-NE; **(D)** Scanning electron microscopy of N-NE; **(E)** Energy dispersive spectrum pattern of E-NE; **(F)** Energy dispersive spectrum pattern of N-NE.

The characterization of nanoemulsions has been investigated using UV-Vis, DLS, zeta potential, TEM, SEM, and EDS. A probe was used to sonicate 100 mL of pre-emulsion by inserting it into the 250 mL glass beaker’s center to a depth of 1.75 cm. The reaction mixture became semitransparent dispersal after 20 min of sonication, indicating the presence of a nanoemulsion with stable physicochemical properties. The mixture displayed a potent absorption peak of E-NE and N-NE at 290 nm and 285 nm, respectively (**Figure 1A**). DLS was used to examine the size distribution of E-NE and N-NE. According to the data in (**Figures 1B, C**), the mean average size is 58.32 ± 1.882 d. nm and 43.29 ± 0.090 d. nm, with PDI of 0.362 ± 0.042 and 0.321 ± 0.091, respectively. The E-NE and N-NE had zeta potential mean values of -4.65 mV and -6.07 mV with a conductivity of 0.00459 ms/cm and 0.0142 ms/cm, respectively (**Figures 1D, E**). TEM and SEM micrographs showed that the synthesized E-NE and N-NE had a spherical structure with a size ranging from 15.01 to 42.24 nm (**Figures 2A, B**), and from 24.02 to 76.45 nm (**Figures 2C, D**), respectively. Based on the EDS analysis of E-NE, the peaks of the elements carbon, oxygen, nitrogen, chloride, and potassium were 51.62, 10.94, 3.03, 15.73, and 18.68%, respectively (**Figure 2E**), whereas N-NE finds the presence of the elements calcium, carbon, and oxygen during examination with values of 24.57, 66.31, and 9.13%, respectively (**Figure 2F**). Even after centrifuging the prepared E-NE and N-NE for 20 minutes at 10,000 rpm at room temperature, they remained stable. No phase separation or creaming was observed after 60 days of storage at various temperatures.

### 3.2 Chemical characterization of nanoemulsions

Gas chromatography and mass spectrometry were used to identify the bioactive chemical components of E-NE and N-NE, and the results showed the existence of 23, and 22 compounds, respectively. Eucalyptol predominated in both E-NE and N-NE in concentrations of 23.19% and 12.32%, respectively. The additional major components in E-NE were D-limonene (28.02%), 1,5-cyclooctadiene,3,4-dimethyl (18.04%), benzene,1-methyl-3-(1 methyl ethyl) (10.69%), and gamma.-terpinene (7.71%), as shown in **Table 1**, while cyclohexane,1-methylene-4-(1 methylethenyl) (65.71%), L-.alpha.-terpineol (10.11%), and cyclohexanol,1-methyl-4-(1methylethylidene) (3.77%) were abundant constituents of N-NE, as got in **Table 2**. Whereas, the remaining chemical components from 23 and 22 in E-NE and N-NE were less than 3%, respectively.

**Table 1:** GC -MS analysis of eucalyptus nanoemulsion.

Compounds	Other Names	RT (min)	Molecular Formula	Relative Peak Area (%) ^a^
Bicyclo [3.1.0] hex-2-ene,2-methyl-5-(1-methylethyl)	3-Thujene	4.2004	C_10_H_16_	0.30
3-carene	Carene	4.3807	C_10_H_16_	2.286
Camphene	Comphene	4.7705	C_10_H_16_	0.60
Beta-phellandrene	β-Phellandrene	5.3101	C_10_H_16_	2.39
Cyclohexane,1-methylene-4-(1-methylethenyl)	Pseudolimonen	5.4160	C_10_H_16_	1.13
Bicyclo[3.1.1]heptane,6,6-dimethyl-2-methylene-,(1S)-	β-Pinene-(1S)- (-)	5.6983	C_10_H_16_	0.65
6-Hepten-1-ol, 2-methyl-	2-Methyl-6-hepten-1-ol	5.7766	C_8_H_16_O	0.04
Alpha. -phellandrene	α-phellanderene	6.0654	C_10_H_16_	0.34
1,3-Cyclohexadiene,1methyl-4-(1- methylethyl)-	α-Terpinen	6.3039	C_10_H_16_	1.73
**Benzene,1-methyl-3-(1methylethyl)-**	**β-Cymene**	**6.4786**	**C_10_H_14_**	**10.69**
N-Dimethylaminomethyl-tert.-butylisopropylphosphine	dimethylaminomethylisopropyl-t-butylphosphine	6.5946	C_10_H_24_NP	0.02
**1,5-Cyclooctadiene,3,4-dimethyl-**	**3,4-dimethyl-1,5-cyclooctadiene**	**6.6041**	**C_10_H_16_**	**18.04**
**D-limonene**	**Cyclohexene**	**6.6053**	**C_10_H_16_**	**28.02**
**Eucalyptol**	**1,8-cineol**	**6.6743**	**C_10_H_18_O**	**23.19**
**Gamma-terpinene**	**γ-Terpinene**	**7.1834**	**C_10_H_16_**	**7.71**
2-Furanmethanol,5ethenyltetrahydro-alpha, alpha,5-trimethyl-, cis-	Linalool oxide	7.4333	C_10_H_18_O_2_	0.08
Cyclohexene,.1-methyl-4-(1 methylethylidene)-	α- Terpinolen	7.7107	C_10_H_16_	1.56
L-fenchone	(R)-Fenchone	7.7699	C_10_H_16_O	0.10
Benzene,1-methyl-3-(1 methylethenyl)-	M-Cymenene	7.7930	C_10_H_12_	0.07
(+)-2-bornanone	Camphor	8.8463	C_10_H_16_O	0.11
Terpinen-4-ol	4-Terpinenol	9.4315	C_10_H_18_O	0.0004
Benzenemethanol,alpha,alpha,4-trimethyl-	P-Cymen-8-ol	9.5294	C_10_H_14_O	0.026
Phenol,2,4-bis(1,1-dimethylethyl)-, phosphite (3:1)	Alkanox 240	30.4358	C_42_H_63_O_3_P	0.13

RT, retention time (minutes). Components present in E-NE at greater than 3% are indicated by bold font. ^a^ Percentages were calculated based on normalized FID peak areas.

**Table 2:** GC-MS analysis of nutmeg nanoemulsion.

Compounds	Other Names	RT (min)	Molecular Formula	Relative Peak Area (%) ^a^
3-carene	Carene	4.3908	C_10_H_16_	0.33
Bicyclo[3.1.0]hex-2-ene,2-methyl-5-(1-methylethyl)	3-Thujene	4.3910	C_10_H_16_	0.29
Camphene	Comphene	4.7825	C_10_H_16_	0.11
Bicyclo[3.1.0]hexane, 4-methylene-1-(1- Methylethyl)-	4-thujene	5.3268	C_10_H_16_	0.35
**Cyclohexane,1-methylene-4-(1 methylethenyl)-**	**Pseudolimonen**	**5.4793**	**C_10_H_16_**	**65.71**
Bicyclo[3.1.1]heptane,6,6-dimethyl-2-methylene-,(1S)-	β-Pinene-(1S)-(-)	5.7112	C_10_H_16_	0.84
Alpha.-phellandrene	α-phellanderene	6.0736	C_10_H_16_	0.30
1,3-Cyclohexadiene,1.methyl-4-(1- methylethyl)-	α-Terpinen	6.3085	C_10_H_16_	0.82
Benzene,1-methyl-3-(1 methylethyl)-	β-Cymene	6.4714	C_10_H_16_	0.63
Alpha-phellandrene	α-phellanderene	6.6013	C_10_H_14_	0.005
Bicyclo[3.1.0]hex-2-ene, 4-methyl-1-(1- Methylethyl)-	2-Thujene	6.6019	C_10_H_16_	0.43
**Eucalyptol**	**1,8-cineol**	**6.6495**	**C_10_H_18_O**	**12.32**
Gamma-terpinene	γ-Terpinene	7.1785	C_10_H_18_O	0.24
3-Cyclohexen-1-ol,1-methyl-4-(1-methylethyl)-	L-4-terpineneol	8.6615	C_10_H_16_	0.28
Cyclohexanol,1-methyl-4-(1-methylethenyl)-	β-Terpineol	8.9011	C_10_H_18_O	1.85
Cyclohexanemethanol,alpha,alpha-dimethyl- 4-methylene-	δ-Terpineol	9.2608	C_10_H_18_O	0.09
Bicyclo[2.2.1]heptan-2-ol,1,7,7trimethyl-, (1S-endo)-	Borneol	9.2896	C_10_H_18_O	0.13
Terpinen-4-ol	4-Terpinenol	9.4361	C_10_H_18_O	0.07
**L-alpha-Terpineol**	**α-Terpieol**	**9.6950**	**C_10_H_18_O**	**10.11**
**Cyclohexanol,1-methyl-4-(1-methylethylidene)-**	**γ-Terpineol**	**9.7685**	**C_10_H_18_O**	**3.77**
Eugenol	Eugenol	12.0267	C_10_H_12_O_2_	0.20
Longifolene	Longifolene	12.8723	C_15_H_24_	0.12
Phenol, 2,4-bis(1,1-dimethylethyl)-, phosphite (3:1)	Alkanox 240	30.4237	C_42_H_63_O_3_P	0.34

RT, retention time (minutes). Components present in N-NE at greater than 3% are indicated by bold font. ^a^ Percentages were calculated based on normalized FID peak areas.

### 3.3 Termiticidal assay

**Figure 3:**
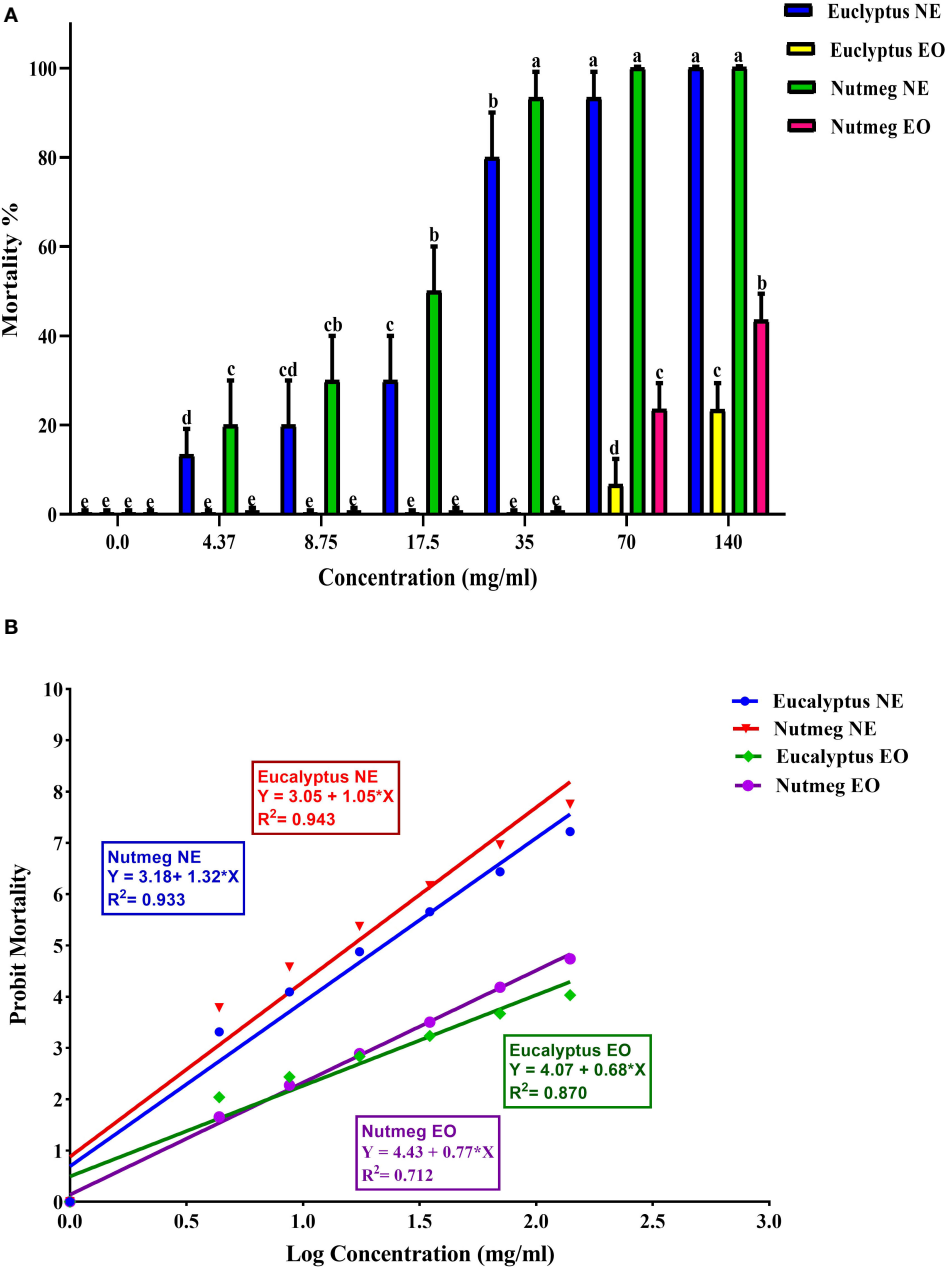
Toxicity effect of eucalyptus and nutmeg nanoemulsions and their essential oil against *O. formosanus* workers 24 h post-treatment at different concentration. **(A)** Percentage of mortality versus concentration; **(B)** Percentage of mortality in probit line unit versus log concentration. Means ± SD values with different letters (a-e) are significantly different at level of *P* < 0.05 according to Tukey’s test.

This study evaluated the effectiveness of EOs and NEs against *O. formosanus* old workers. The results showed that within 24 h of treatment, *O. formosanus* was suppressed by E-NE and N-NE at different concentrations, and the mortality rate increased with increasing concentration. The mortality rates for E-NE and N-NE were 100% and 99.53%, respectively with the highest concentration (140 mg/mL). In contrast, with the same concentration, the mortality rates for E-EO and N-EO were 23.43% and 43.55%, respectively (**Figures 3A, B**). This indicates that when compared E-EO and N-EO with E-NE and N-NE were more effective in suppressing *O. formosanus*. In addition, LC_50_ and LC_90_ of E-EO, N-EO, E-NE, and N-NE were evaluated (**Table 3**). The results showed that the LC_50_ of E-EO, N-EO, E-NE, and N-NE were 332.96, 157.58, 19.52, and 12.62 mg/mL, respectively. While the LC_90_ of E-EO, N-EO, E-NE, and N-NE were 2094.95, 488.70, 60.91, and 38.65 mg/mL, respectively. These results indicated that E-NE and N-NE were more toxic than E-EO and N-EO and N-NE was more toxic to *O. formosanus*.

**Table 3:** Toxicity of eucalyptus and nutmeg nanoemulsions and their essential oils against *O. formosanus* workers 24 h post-treatment.

Treatment	Concentrations (mg/ml)	Mortality (%) ± SD	LC_50_ (95% LCL-UCL)	LC_90_ (95% LCL-UCL)	X^2^
**E-NE**	0.0	0.0 ± 0.0^e^	19.52 (12.91-29.00)	60.91 (38.72-149.95)	1.83
	4.37	13.3± 5.7^d^			
	8.75	20± 10^cd^			
	17.5	30± 9.9^c^			
	35	80± 10^b^			
	70	93.3 ± 5.7^a^			
	140	100 ± 0.23^a^			
**E-EO**	0.0	0.0 ± 0.0^e^	332.96.(126.45- 4.307E+12)	2094.95.(361.08 6.111E+24)	0.352
	4.37	0.33 ± 0.57^e^			
	8.75	0.33 ± 0.57^e^			
	17.5	0.33 ± 0.57^e^			
	35	0.33 ± 0.57^e^			
	70	6.66 ± 5.77^d^			
	140	23.43 ±5.94^c^			
**N-NE**	0.0	0.0 ± 0.0	12.62 (8.00-18.62)	38.65 (24.82-97.65)	2.26
	4.37	20 ± 5.05^c^			
	8.75	30 ± 5.7^bc^			
	17.5	50 ± 2.02^b^			
	35	80.02 ± 2.46^a^			
	70	96.23 ± 5.46^a^			
	140	99.53 ± 1.05^a^			
**N-EO**	0.0	0.0 ± 0.0^e^	157.58 (96.9-1566.5)	488.70 (208.50-162913.90)	5.21
	4.37	0.77 ± 0.69^e^			
	8.75	0.77 ± 0.69^e^			
	17.5	0.77 ± 0.69^e^			
	35	0.77 ± 0.69^e^			
	70	23.54 ± 5.8^c^			
	140	43.55 ± 5.8^b^			

LC_50_, lethal concentration that kills 50% of insects; LC_90_, lethal concentration that kills 90% of insects; LCL, lower confidence limit; UCL, upper confidence limit; SD, standard deviation; EO, essential oil; and NE, nanoemulsion; X^2^ Chi-squared values at different df and probability level (0.05).

### 3.4 Biochemical assay

#### 3.4.1 Effect of nanoemulsions on enzymatic activities in the termites

**Figure 4:**
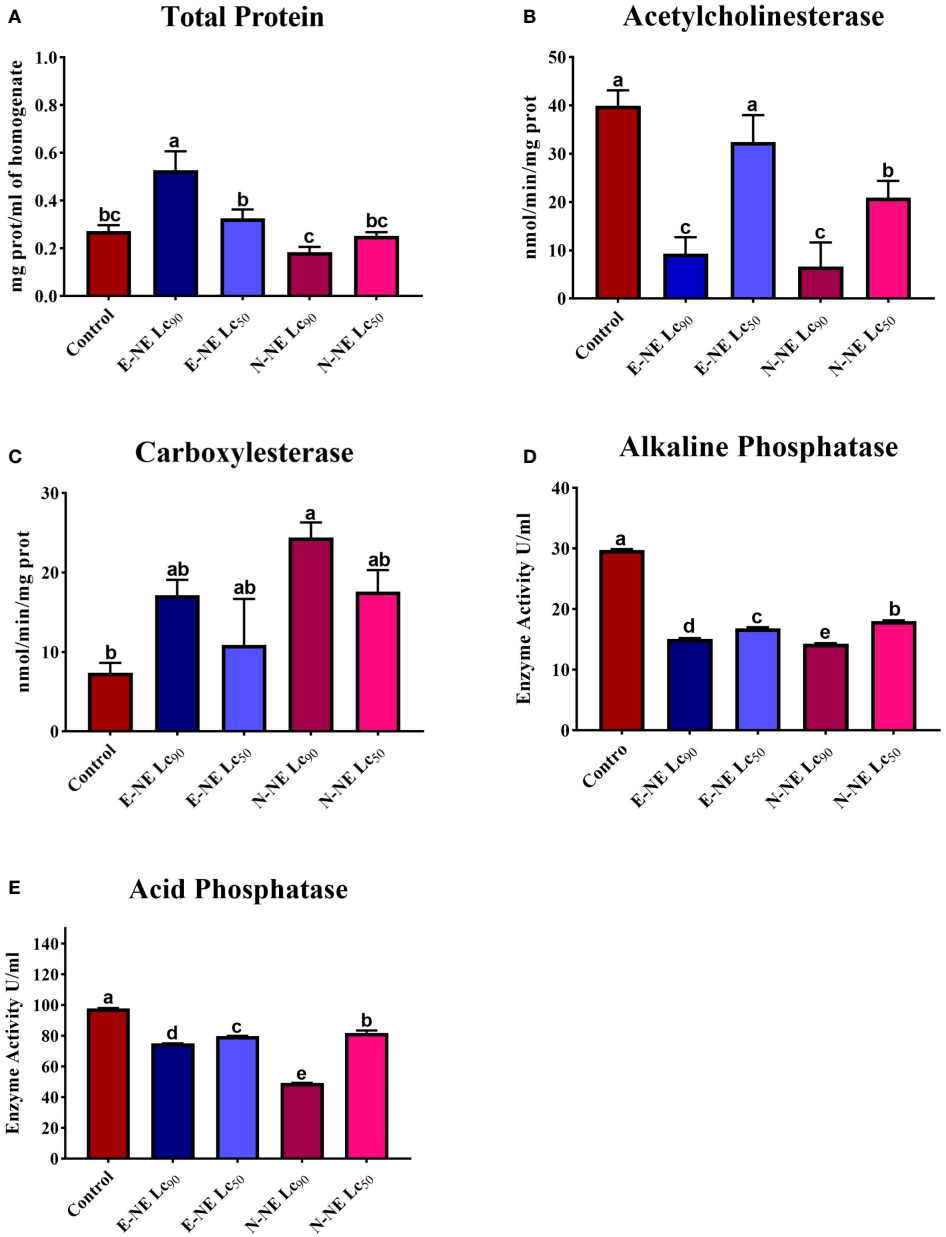
Effects of eucalyptus and nutmeg NEs on the total protein level and enzyme activities in termite workers. **(A)** Total protein concentration; **(B)** Acetylcholinesterase; **(C)** Carboxylesterase; **(D)** Alkaline phosphatase; **(E)** Acid phosphatase. Different letters above the bars of each figure indicate significant differences based on Tukey’s test at *P*< 0.05 between control and other treatments. Each bar represents the mean ± SE of three replicates using different preparations of insect homogenates.

According to the above results, the efficiency of E-NE and N-NE in suppressing *O. formosanus* was higher than that of E-EO and N-EO. Therefore, the effectiveness of E-NE and N-NE as termiticides against *O. formosanus* was evaluated by studying their effect on enzymes involved in biological processes in termites, such as acetylcholinesterase activity, carboxylesterase activity, acid phosphatase activity, alkaline phosphatase activity, as well as total protein levels. Compared to the control group, LC_50_ and LC_90_ of E-NE and N-NE caused differences in the natural biochemical components of the old workers, either increasing or decreasing their activity as shown in **Figure 4**. For the total protein concentration assay, compared to the control, the total protein concentration significantly increased after exposure to E-NE at LC_50_ and LC_90_. Where the total protein concentration in the control group was 0.27 mg/mL, while 0.32 and 0.52 mg/mL, respectively, of E-NE at LC_90_ and LC_50_. In contrast, exposure to N-NE exhibited a significantly decreased level of total protein. Where the total protein concentration was 0.25 and 0.18 mg/mL, respectively, of N-NE at LC_50_ and LC_90_ compared with the control (**Figure 4A**). Furthermore, compared with the control, the treatment with E-NE and N-NE at LC_90_ significantly decreased AChE activity from 39.95 nmol/min/mg prot to 9.25 nmol/min/mg prot and 6.62 nmol/min/mg prot, respectively. Whereas E-NE and N-NE at LC_50_ showed a slight reduction in the activity of AChE with values of 32.44 nmol/min/mg prot and 20.89 nmol/min/mg prot, repectively (**Figure 4B**). These results suggest that the LC_50_ and LC_90_ of E-NE and N-NE caused a decrease in AChE in treated termite workers. The results indicated that the old workers’ *O. formosanus* treatment with E-NE and N-NE at LC_50_ and LC_90_ increased CarE activity compared to the untreated group. While the CarE concentration in the control group was 7.37 nmol/min/mg, the concentration of CarE was 10.92 nmol/min/mg and 17.13 nmol/min/mg, respectively, for workers treated with E-NE at LC_50_ and LC_90_, and 17.59 nmol/min/mg and 24.41 nmol/min/mg, respectively, for N-NE-treated workers at LC_50_ and LC_90_ (**Figure 4C**). Therefore, the results of this study indicate that E-NE and N-NE at LC_50_ and LC_90_ were highly toxic in killing and suppressing *O. formosanus*. On the contrary, alkaline phosphatase activity significantly decreased in workers treated with E-NE and N-NE at LC_50_ and LC_90_ compared with the control group. While the alkaline phosphatase concentration in the control group was 29.74 U/mL, the concentration of alkaline phosphatase was 16.84 U/mL and 15.11 U/mL, respectively, for workers treated with E-NE at LC_50_ and LC_90_ and 18.04 U/mL and 14.27 U/mL for N-NE-treated workers at LC_50_ and LC_90_ (**Figure 4D**), also there was a significantly decreased in acid phosphatase activity compared with the control group value of 97.99 U/mL to 79.93 U/mL and 75.12 U/mL, respectively, of E-NE at LC_50_ and LC_90_, and 81.91 U/mL and 49.28 U/mL respectively, of N-NEs at LC_50_ and LC_90_ (**Figure 4E**).

### 3.5 Morphological observations post-exposure to nanoemulsions

**Figure 5:**
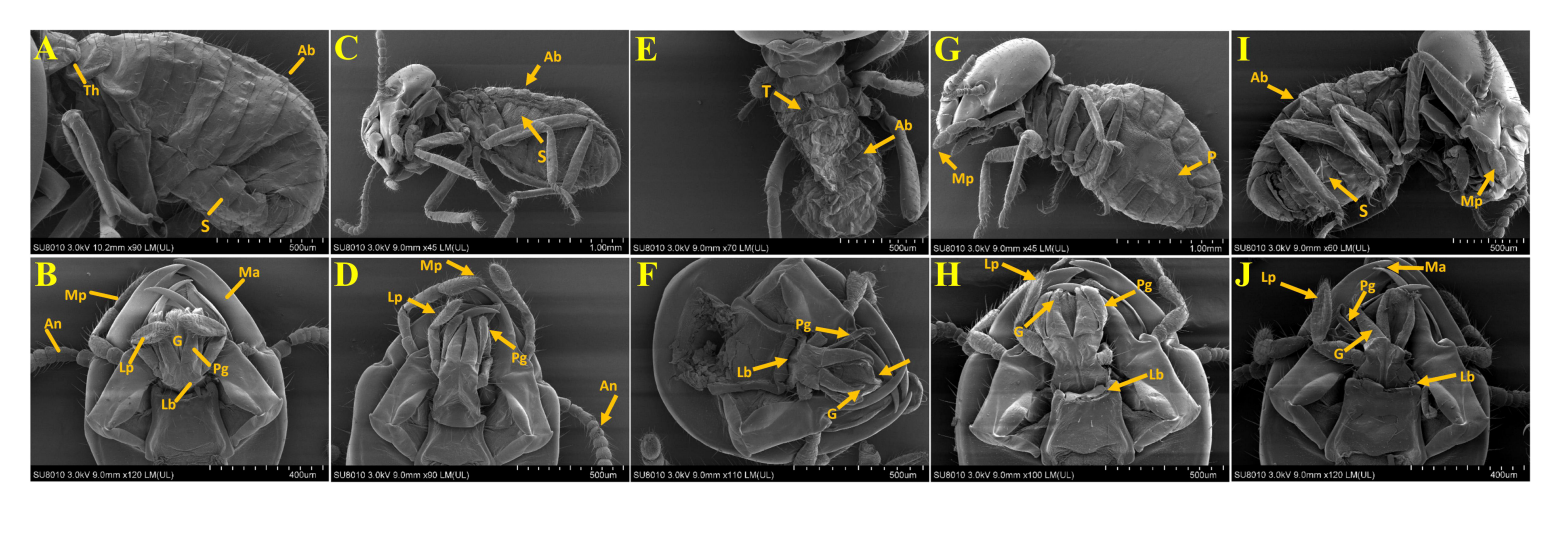
Morphological study of *O. formosanus* workers post-exposure to eucalyptus and nutmeg NEs. **(A, B)** Control; **(C, D)** E-NE at LC_50_; **(E, F)** E-NE at LC_90_; **(G, H)** N-NE at LC_50_; **(I, J)** N-NE at LC_90_. Th, Thorax; Ab, Abdomin; An, Antannae; P, Pleura; S, Sternum; T, Tergum; Ma, Mandibule; Mp, Maxillary palp; Lb, Labium; Lp, labial palp; G, Glossa; Pg, Paraglossa. Arrow indicates damages in *O. Formosanus* workers.

The effects of E-NE and N-NE at LC_50_ and LC_90_ on the morphological changes of *O. formosanus* worker termites are shown in **Figure 5**. The SEM images showed that E-NE and N-NE of LC_50_ and LC_90_ induced significant changes in morphology in different parts of the body of *O. formosanus*. Also, E-NE and N-NE’s LC_90_ had a more severe effect than the LC_50_’s, dealing massive damage. In treated termites, the deformities are shrunken in the cuticles of the pleura, sternum, and tergum and damage to various parts of the mouthparts, such as the mandible, labium, glossa, paraglossa, labial palp, maxillary palp, and clypeus. On the other hand, no morphological changes occurred in the untreated termites, and all parts appeared intact. Therefore, these results indicate that E-NE and N-NE played a significant role in suppressing *O. formosanus*.

### 3.6 Histological observations post-exposure to nanoemulsions

**Figure 6:**
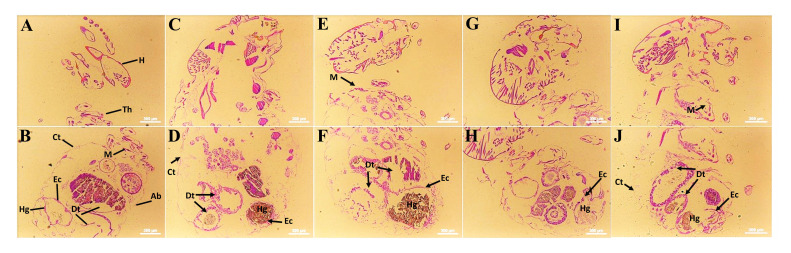
Histological study of *O. formosanus* workers post-exposure to eucalyptus and nutmeg NEs. **(A, B)** Control; **(C, D)** N-NE at LC_50_; **(E, F)** N-NE at LC_90_; **(G, H)** E-NE at LC_50_; **(I, J)** E-NE at LC_90_. H, Head; Th, Thorax; Ab, Abdomin; Ct, Cuticle; Hg, Hind gut; M, Muscles; Dt, Digestive tract; Ec, Epithelial cells. Arrow indicates damages in *O. Formosanus* workers.

The histological results in workers of *O. formosanus* treated with E-NE and N-NE for 24 h at LC_50_ and LC_90_ are shown in **Figure 6**. In comparison with control, termites exposed to E-NE and N-NE, especially at LC_90_ revealed various histological structure alterations in the cuticles, muscles, and digestive tract especially hind gut, while at LC_50_ slight changes were observed in the same areas. These studies noted that epithelial cells of the digestive tract reduced intercellular contacts with neighboring cells, and detachment cells from the basal lamina, in addition to degeneration of cells after treatment, while control workers exhibited a normal appearance of the entire body regions (head, thorax, and abdomen) and regular cells. There is also leakage in the gut contents.

## 4 Discussion

Uncontrolled usage of traditional insecticides has produced resistant insects with various modes of action. In addition, using insecticides has a detrimental effect on biocontrol agents, promotes the reappearance of secondary pests, and leaves environmental residues. This is why finding and creating fresh alternatives to traditional methods of managing insects with economic value has been deemed crucial. These new tactics ought to be efficient, biodegradable, less harmful, and aggressive in terms of the environment (Senthil-Nathan, 2020). Insecticides made from natural ingredients are one of the alternatives that have been taken into consideration for the development of new pesticides. Plant extracts and essential oils have been highlighted because their chemical compositions reflect the natural coevolutionary interactions between plants and insects (Pavela, 2015). Making nanoemulsions from essential oils enhances the efficacy of insecticides by allowing better dispersion of their constituents in an aqueous medium (Sharma et al., 2020b; Folly et al., 2021; Martins et al., 2021).

This is the first time that a nanoemulsion formulation using E-EO and N-EO has been reported against *O. formosanus*. The present study focuses on the synthesis and characterization of E-NE, and N-NE and studies their effective termiticides, biomedical properties, and morpho-histological properties. The formation of E-NE and N-NE was confirmed through UV–Vis spectrophotometry, showing strong absorption peaks at 290 nm and 285 nm, respectively. The same range in UV absorbance was reported in previous studies of other nanoemulsions of essential oils, for instance, *Melaleuca alternifolia* (Maiden & Betche) Cheel (*M. alternifolia*) (Ibrahium et al., 2022), *Ocimum basilicum* L. (*O. basilicum*), and *Cuminum cyminum* L. (*C. cyminum*) (Abdelaal et al., 2021). Our findings show that E-NE and N-NE have small particle sizes and PdI values below 0.04, which show that the NE droplets are monodispersed, have a limited size distribution, and have good long-term stability (Barik and Singh, 2019). Our results show the stability of both nanoemulsions after storage for 60 days. According to Prateeksha et al. (2019) even after centrifuging the *Gaultheria fragrantissima* Wall nanoemulsion for 20 minutes at 10,000 rpm, it remained stable. After being kept for 3 months at different temperatures. Additionally, the study’s Zeta potential means were far than zero, which suggests that the nanoemulsion is still stable because values close to zero signify flocculation (Pinto and Buss, 2020). According to Adak et al. (2020), high values of zeta potential provide a low sedimentation rate and prevent a quick phase separation, which can be described by the Brownian motion. This preserved the emulsion stability in the current investigation, which was connected with nanometric sizes and low PDI values. The electrical conductivity of the nanoemulsions was measured to evaluate their stability and the nature of the formulation. It provides information about nanoemulsions continuous phase and phase inversion phenomena. Conductivity is the ability of any molecule to transfer electricity between two points (Talegaonkar et al., 2011). Ibrahium et al. (2022), recorded that nanoemulsions of *M. alternifolia* and *Citrus limon* (L.) Osbeck have mean droplet sizes of approximately 188.4 d.nm and 50.66 d.nm with PDI values of 0.527 and 0.171, respectively, and a Z-average of -2.02 mV and -2.30 mV, respectively. Clerici et al. (2018), detected DLS for *E. globulus* nanoemulsion was 79.80 ± 0.37 nm with a PDI value of 0.21 ± 0.007. The testing yielded negative results for the zeta potential. TEM image of E-NE and N-NE highlighted that they were spherical in shape, with a size ranging from 15.01 to 42.24 nm, and from 24.02 to 76.45 nm, respectively. which was consistent with the results of the eucalyptus nanoemulsion droplets, done by Sharma et al. (2020a) were almost spherical in shape; also, Kumari et al. (2018), revealed spherical droplets of 80-150 nm in thymol nanoemulsion. In this study, EDS analysis of E-NE and N-NE recorded several element compositions. In accordance with Abdelaal et al. (2021), the main components of *O. basilicum* nanoemulsions are calcium, copper, zinc, potassium, and magnesium.

The exact composition and proportion of the eucalyptus oil and nutmeg oil vary with species and are made up of a complex mixture of different monoterpenes and sesquiterpenes, as well as oxides, aromatic phenols, ethers, alcohols, aldehydes, esters, and ketones (Saulle et al., 2018). The pesticidal activity of essential oils has been because of the components such as eucalyptol, 3,7-dimethyl-, citronellol, 3,7-Dimethyloct-6-en-1-yl acetate, p-cymene, eucamalol, limonene, linalool, γ-terpinene, α-pinene, and α-terpineol (Liu et al., 2008). The chemical compositions of E-NE and N-NE were identified by GC-MS. The main chemicals found in E-NE are D-limonene, eucalyptol, 1,5-cyclooctadiene,3,4-dimethyl, benzene,1-methyl-3-(1 methylethyl), and gamma-terpinene, while cyclohexane,1-methylene-4-(1 methylethenyl), eucalyptol, L-alpha-terpineol, and cyclohexanol,1-methyl-4-(1methylethylidene) are the main constituents at N-NE. Sugumar et al. (2014) reported that eucalyptol represents 17.73% of eucalyptus oil, which is the primary component of the oil. Whilst Adak et al. (2020) recorded that the major ingredients of eucalyptus oil were eucalyptol (64.80%), α-pinene (11.17%), β-pinene (8.19%), γ-terpinene (5.91%), and α-phellandrene (3.88%). Meanwhile, β-pinene (26%), α-pinene (10.51%), sabinene (9.16%), and γ-terpinen (8.51%) were the primary components of nutmeg oil (Cossetin et al., 2021). As mentioned by another study, the main substances of nutmeg oil are α-pinene with 7.17%, γ-terpinen with 19.08%, terpinolene with 2.06%, and myristicin with 7.33% (Agustinisari et al., 2013). The constituents can differ depending on the species, season, and crop growing conditions. The process of forming nanoemulsions does not alter the compounds contained in oil; only the concentration is changed because only a specific amount is required to emulsify the oil.

The maximum mortality was seen, especially for E-NE and N-NE, which had LC_50_ and LC_90_ values of 19.52 and 12.62 mg/mL and 60.91 and 38.65 mg/mL, respectively, and were more potent than essential oils. According to Massoud et al. (2018), the smaller particle size and larger surface area of emulsion droplets in NEs could guarantee formulation interaction with the target pest and improve penetration through the insect cuticle. A similar result was reported by Adak et al. (2020), who utilized eucalyptus nanoemulsion against *S. oryzae* and *T. castaneum.* There are several studies of nanoemulsions of various essential oils against many aphid species and other insect pests compared with their bulk forms. For instance, NE of *O. basilicum*, *C. cyminum*, *Origanum marjorana* L., and *Matricaria chamomilla* L. proved considerable toxic activity against *Aphis craccivora* (C.L.Koch, 1854) (*A. craccivora*) (Abdelaal et al., 2021). The larvicidal activity of eucalyptus nanoemulsions against *Culex quinquefasciatus* (Say, 1823) (*C. quinquefasciatus*) was found to be more effective than its bulk counterpart (Sugumar et al., 2014).

In this regard, it has been demonstrated that numerous plant compounds used to manage insect pests affect the enzyme profiles of insects (Smirle et al., 1996; Zhang et al., 2013). This can attest to the phytochemicals in essential oils’ ability to pierce insects’ defense mechanisms and affect their enzymatic activity, leading to physiological dysfunction (Czerniewicz et al., 2018). Protein synthesis is a crucial biological function in every cell and living organism because it is the fundamental component of tissues (Avila et al., 2011). In accordance with earlier studies done by Koodalingam et al. (2012), there was a decrease in the protein concentration of the larvae of *Aedes aegypti* (Linnaeus in Hasselquist, 1762) (*A. aegypti*) on exposure to a Bt-based product, which is in agreement with the result of the current study. This study demonstrates a significant reduction in AChE activity after exposure to E-NE and N-NE. This could simply be attributed to the presence of some terpenoids in the tested formulations. Moreover, this reduction could impair neuromuscular coordination, eventually resulting in paralysis and death (Lu et al., 2013). Yang et al. (2021) discovered that *Reticulitermes dabieshanensis* (Wang & Li, 1984) treated with different nanoemulsions showed a reduction of AChE activity, which is consistent with our work. In addition, *C. quinquefasciatus* larvae exposed to a eucalyptus nanoemulsion showed a significant reduction in AChE activity (Sugumar et al., 2014). Esterases are among the detoxifying enzymes that merit greater discussion since they can be involved in the metabolism of a wide range of endogenous and exogenous substances (Li et al., 2007). CarE is an enzyme responsible for insect detoxification, and its level increases with the level of toxicity to which the insect is exposed. Consistent with our findings, CarE activity was significantly increased in *Helicoverpa armigera* (Hübner, 1808) larvae after exposure to quercetin (Chen et al., 2017). Adult *Rhyzopertha dominica* (Fabricius, 1792) (*R. dominica)* given with a sublethal dosage of eucalyptus essential oils showed increased α- and β-esterase activity (Ebadollahi et al., 2022b). The α- and β-esterase activity of adult *R. dominica* treated with lethal and sublethal doses of thymus essential oils was elevated, which is consistent with our results (Ebadollahi et al., 2022a). It should be highlighted, nevertheless, that the essential oils work through a variety of mechanisms, including disruption of the octopamine receptors and inhibition of acetylcholinesterase and glutathione -*S*- transferases (Regnault-Roger et al., 2012; Isman, 2020). Acid and alkaline phosphatases are vital in the hydrolytic cleavage of phosphoric acid esters and in regulating the acid-alkali balance (Walter and Schuett, 1974). These enzymes play a crucial role in other crucial physiological functions as well, including metabolism and cellular interaction (Nathan et al., 2007). According to earlier studies, the levels of acid and alkaline phosphatase in *C. quinquefasciatus* larvae were shown to decrease after treatment with eucalyptus oil nanoemulsion (Sugumar et al., 2014). The alkaline and acid phosphatase enzymes were decreased in comparison with the control in the larvae of *Cnaphalocrocis medinalis* (Guenée, 1854) when treated with plant secondary metabolites, which was consistent with our findings (Nathan et al., 2007). There is a decrease in ALKP and ACP activity in termites treated with E-NE and N-NE.

The SEM observations of morphological changes in *O. formosanus* workers confirm that the E-NE and N-NE may operate in the cuticle to affect motility, development, and lethality. The SEM results were not compared to the earlier investigations because there isn’t much information in the literature regarding how nanoemulsions affect *O. formosanus*. Nevertheless, prior research on the structural abnormalities brought on by *Tagetes minuta* L. oil against *A. craccivora*, and *Plutella xylostella* (Linnaeus, 1758) was scarce and substantially corroborated the current study’s findings (Dolma et al., 2022; Jayaram et al., 2022). Also the ocimene-treated *Planococcus lilacinus* (Cockerell, 1905) showed multiple abnormalities on the setae, thoracic leg, and cuticle (body fluid accumulation and thickness encrustations) (Arokiyaraj et al., 2022). According to findings from other authors (Botas et al., 2017; Pessoa et al., 2018), *A. aegypti* and *C. quinquefasciatus* larvae showed modifications in the cuticle of the head, thorax, abdomen, and siphon when treated with various nanoemulsions. The histological assays of E-NE and N-NE towards workers of *O. formosanus* showed that the cuticles, muscles, and epithelial cells are the most affected tissues in termites. According to Sugumar et al. (2014), *C. quinquefasciatus* larvae show damaged peritrophic membranes, damaged midgut epithelial cells, and leakage of the midgut’s contents after exposure to eucalyptus oil nanoemulsion. The mid gut region of the insect is an important region that is linked to the release of digestive enzymes and nutrition absorption. Additionally, it is possible to investigate the integrity of the mid gut as a sensitive indicator of toxicity toward a number of poisonous substances (Sutherland et al., 2002).

## 5 Conclusion

To the best of our knowledge, this is the first time nanoemulsions of eucalyptus and nutmeg essential oils have been synthesized to control *O. formosanus*. The nanoemulsion formulation from eucalyptus and nutmeg essential oils ensures higher efficacy as a termiticidal agent against *O. formosanus* when compared to its bulk essential oils. Furthermore, our results exhibit insights into the mechanism of action of eucalyptus and nutmeg nanoemulsions on workers of *O. formosanus*, both of which influence enzymatic pathways and morpho-histological structures. Eucalyptus and nutmeg essential oils, being plant-based components, are eco-friendly and have natural pesticide activity. From this study, it can be concluded that eucalyptus nanoemulsion and nutmeg nanoemulsion can be utilized as a safe and effective alternative in the management of Subterranean termites, *O. formosanus*, as a safe substitution for hazardous chemical pesticides.

## Data availability statement

The original contributions presented in the study are included in the article/Supplementary Material. Further inquiries can be directed to the corresponding author.

## Ethics statement

The manuscript presents research on animals that do not require ethical approval for their study.

## Author contributions

RN: Conceptualization, Formal analysis, Software, Writing – original draft. EI: Investigation, Writing – review & editing. HF: Writing – review & editing, Data curation. FA: Writing – review & editing, Visualization. WL: Methodology, Resources, Writing – review & editing. QZ: Resources, Writing – review & editing. TY: Resources, Writing – review & editing. NC: Visualization, Writing – review & editing. JM: Funding acquisition, Project administration, Supervision, Writing – review & editing.
